# Kurdish News Dataset Headlines (KNDH) through multiclass classification

**DOI:** 10.1016/j.dib.2023.109120

**Published:** 2023-04-13

**Authors:** Soran Badawi, Ari M. Saeed, Sara A. Ahmed, Peshraw Ahmed Abdalla, Diyari A. Hassan

**Affiliations:** a Language Center, Charmo University, KRG, Chamchamal, Kurdistan, Iraq; b Computer Science Department, University of Halabja, KRG, Halabja, Kurdistan, Iraq; c Department of Computer Science, Komar University of Science and Technology, Sulaymaniyah, Kurdistan Region, Iraq; d Faculty of Engineering & Computer Science, Qaiwan International University, Sulaymaniyah, Kurdistan Region-Iraq

**Keywords:** Kurdish text classification, News headline dataset, Natural language processing, Text pre-processing

## Abstract

The rapid growth of technology has massively increased the amount of text data. The data can be mined and utilized for numerous natural language processing (NLP) tasks, particularly text classification. The core part of text classification is collecting the data for predicting a good model. This paper collects Kurdish News Dataset Headlines (KNDH) for text classification. The dataset consists of 50000 news headlines which are equally distributed among five classes, with 10000 headlines for each class (Social, Sport, Health, Economic, and Technology). The percentage ratio of getting the channels of headlines is distinct, while the numbers of samples are equal for each category. There are 34 distinct channels that are used to collect the different headlines for each class, such as 8 channels for economics, 14 channels for health, 18 channels for science, 15 channels for social, and 5 channels for sport. The dataset is preprocessed using the Kurdish Language Processing Toolkit (KLPT) for tokenizing, spell-checking, stemming, and preprocessing.

**Specifications Table**

**Table:** 

Subject:	Applied Machine Learning
Specific subject area:	Kurdish News Dataset Headlines (KNDH) through Multiclass Classification.
Type of data:	Text Figure Table
How the data were acquired:	ParsHub tool and BeautifulSoup library in Python are used to collect data from news websites.
Data format:	Raw
Description of data collection:	The specific URL is added to the page intended to collect data, and then some headlines are selected for fetching the texts.
Data source location:	Charmo University
Data accessibility:	+Repository name: Mendeley Data Data identification number: 10.17632/kb7vvkg2th.2 Direct link to the dataset [1]: https://doi.org/10.17632/kb7vvkg2th.2

## Value of the Data

1. This is an attempt to build a huge multi-categorical dataset for the Kurdish language. Moreover, it can be beneficial for improving the sentiment analysis field in the Kurdish language.
2. The data include the headlines of popular Kurdish news websites, which researchers can use to conduct research in the language at a syntactical level.
3. Various algorithms can be applied to predict different models in text classification.
4. This dataset provides another reference for the Kurdish language, making it closer to being resourced.
5. Each news website organizes its articles into categories before publishing them, allowing users to quickly select the categories of news that interest them whenever they visit the site. For instance, some readers want to read about the most recent technological advancements, so they always click on the technology section when they visit a news website. They might be interested in politics, business, entertainment, or even sports, but they may not enjoy reading about technology. Currently, due to the need for datasets, the content administrators of news websites manually categorize Kurdish news articles. However, they can also use this dataset to build a highly accurate model, since KNDH is a massive Kurdish dataset for news classification based on five categories, to deploy a machine learning model on their websites that reads the news headline or the news content and identifies the category of the news.

## 1 Objective

The Kurdish language is classified as less resourced in terms of natural language processing (NLP). The similar datasets for other languages previously were conducted, but the sources for the Kurdish languages is inferior and a small number of the dataset available related to the language [2,3]. The language needs essential tools such as name recognition, lemmatization, POS tagger, etc. This issue is primarily rooted in need for a more efficient corpus. The datasets available in the language include collecting comments and tweets from social media. The primary issue regarding these datasets is that they contain many grammatical and dictation errors. Since the language does not have an excellent tool to preprocess those data, the datasets need to be cleaned or require manual preprocessing. To better understand the syntactic and semantic nature of the Kurdish language and have an adequate dataset, our research group collected texts from news headlines written by academic people and contained small numbers of errors. The dataset is suitable for performing text classifications and achieving satisfactory results.

## 2 Data Description

The Kurdish language belongs to the Indo-Iranian family of Indo-European languages. It is well-known to be a close relative to the Persian language. The speakers span the intersections of Iran, Turkey, Iraq, and Syria. The Kurdish language is one of the official languages in Iraq and has regional status in Iran. The language has 40 million speakers [4]. Central Kurdish (Sorani) and Northern Kurdish (Kurmanji) are two of the main dialects of the Kurdish language [5]. However, there are other minor dialects, such as Gorani (Hawrami), spoken in some residential settings in Iraq and Iran, and Zazaki, which is used in Turkey [6]. Historically, many styles of the alphabet have been used for writing Kurdish, namely Cyrillic, Armenian, Latin, and Arabic. The dataset is the Sorani dialect which has 36 letters as vowels and constants [7], as shown in Table 1.

**Table 1:** Kurdish vowels and consonant Letters (Sorani Dialect).

Constants Letters	Constants Letters in Latin	Vowels Letters	Vowels Letters in Latin
ئ	a	ا	a
ب	b	ه	e
پ	p	و	U
ت	t	ۆ	O
ج	c	وو	ừ
چ	ç	ى	i
ح	h’	ێ	ȇ
خ	x		
د	d		
ر	r		
ڕ	rr		
ز	z		
ژ	j		
س	s		
ش	ş		
ع	ë		
غ	x		
ف	f		
ڤ	v		
ق	q		
گ	g		
ک	k		
ل	l		
ڵ	ll		
م	m		
ن	n		
ه	h		
و	w		
ى	y		

The letters in this language do not have capitalization forms, which are written starting from the right-hand side [8]. The Sorani dialect is distinguished by its lack of gender. In Sorani's writing, possessive pronouns, definiteness markers, enclitics, and postpositions are used every time they are inserted as suffixes [9,10]. Furthermore, it contains two tenses, past and present, and singular and plural cases, but with complex morphology [11]. As for the future, the language benefits from its auxiliary verbs to denote actions that will occur in this period. The language is highly inflectional due to many affixes and clitics [12]. Jugal (2014) states that Sorani does not apply gender or grammatical case for its nominals. Although, it has an entire article marking system for definite, indefinite, and demonstrative in singular and plural forms [13]. Regarding Verb, Kurdish has around 300 single-word verbs, which are inflected based on the personal pronouns, which include (first (singular-plural), second (singular-plural), third (singular-plural)), tense(past, present, future), aspect(indefinite, perfect, progressive, imperfective), and mood (indicative, subjunctive, conditional) [14]. The Kurdish language employs compound construction forms to produce new vocabulary, namely (Noun + Verb), (Adjective + Verb), and (Preposition + Verb) forms [15], as shown in Table 2.

**Table 2:** Compound Verb construction (Sorani Dialects).

No	Construction	Example	Translation
1	Noun + Verb	نان + کردن = نان کردن	To make bread
2	Adjective + Verb	پاک + کردن = پاکردن	To purify
3	Preposition + Verb	هەڵ + کردن = هەڵکردن	To turn on

Regarding syntax, the Kurdish language follows subject-object-verb (SOV) order. Since the language is a pro-drop or null subject, thus, the removal of the subject in a sentence will create zero effects on its meaning [16]. The instances are explained as shown in Table 3:

**Table 3:** Samples of Kurdish Sentences (Sorani Dialects).

No	Sentence with Subject	Sentence without subject
1	من ئازادیم خۆش دەوێت (I love freedom)	ئازادیم خۆش دەوێت (I love freedom)
2	ئازاد و ئارام گوڵەکانیان ئاودا (Azad and Aram watered the flowers)	گوڵەکانیان ئاودا (they watered the flowers)
3	کوڕەکە مۆبایلەکەی فرۆشت (the boy sold the mobile)	مۆبایلەکەی فرۆشت (he sold the mobile)

It can be seen that in Table 3, The words (“من”, “ئازاد و ئارام”, and “کوڕەکە”) serve as subjects in the examples. Once they are omitted from the sentences, the meaning of the sentences has not been affected by their removal. Due to this case, the Kurdish language is recognized as a null subject language.

## 3 Data Collection

Technological advancements today have made it possible for news to spread worldwide. News agencies have to cover many things daily due to the tremendous change in the world's state. News in various categories is bombarded on the internet. Every news agency broadcasts, reports or writes fancy headlines to incite users when an incident occurs worldwide. Designing a model which can categorize the news headline is an essential step. An excellent dataset is required to train such a model. In this study, the total number of headlines is 50,000. Samples were collected from many websites such as Rudaw, Payam, Knnc, Kurdsat, etc. The samples are equally distributed across five classes (social, sport, science, health, and economy), as shown in Table 4. The Number and Percentage of Collected Data according to the *channels are shown in* Table 5*.*

**Table 4:** Number and Percentage of Collected Data according to the categories.

Class	No. of Samples	No. of Channels	Channels	Percentage

economic	10.000	8	GaliKurdstan (www.gksat.tv)	3.61%
			K24 (www.kurdistan24.net/ckb)	62.18%
			Kurdistantv (https://kurdistantv.net)	16.85%
			Kurdstat (www.kurdsat.tv)	3.47%
			Payamtv (www.peyam.net)	2.71%
			Rudaw (www.rudaw.net/sorani)	0.13%
			Xendan (www.xendan.org)	4.41%
			Xelk (https://xelk.org)	6.64%
Health	10000	14	Government (https://gov.krd)	1.06%
			K24 (www.kurdistan24.net/ckb)	17.91%
			Kurdistantv (https://kurdistantv.net)	12.91%
			Kurdstat (www.kurdsat.tv)	2.22%
			Payamtv (www.peyam.net)	3.61%
			Jamawar (www.jamawarnews.com)	0.19%
			Knnc (www.knnc.net)	2.42%
			Xelk (https://xelk.org)	3.51%
			Kurdiu (www.kurdiu.org/ku)	14.21%
			Politic press (https://politicpress.com)	20.67%
			Rachlaken (www.rachlaken.com)	2.22%
			Sharpress (www.sharpress.net)	1.49%
			Wishe (www.wishe.net)	10%
			Xwakurk (www.xwakurk.com)	7.52%

Science	10000	18	Al-ashraq (https://aleshraqtv.iq)	3.12%
			Bn24 (https://bn24.org)	0.22%
			Chawder (https://chawder.org)	0.91%
			GaliKurdstan (www.gksat.tv)	3.61%
			K24 (www.kurdistan24.net/ckb)	1.78%
			Khaktv (www.khaktv.com)	5.53%
			Kitn (https://kitn.net)	3.43%
			Kurdistantv (https://kurdistantv.net)	6.82%
			Millatpress (www.milletpress.com)	2.39%
			NRT (www.nrttv.com)	4.69%
			Payamtv (www.peyam.net)	2.73%
			Politic press (https://politicpress.com)	16.75%
			Shanpress (www.shanpress.com)	0.99%
			Westga (https://westganews.net)	6.67%
			Xelk (https://xelk.org)	3%
			Xendan (www.xendan.org)	8.82%
			Xwakurk (www.xwakurk.com)	23.02%
			Zagros (https://zagrosn.com)	5.52%

Social	10000	15	Awene (https://www.awene.com)	1.81%
			Dwaroj (https://dwaroj.net)	0.21%
			Government (https://gov.krd)	0.48%
			Gulan (www.gulanmedia.com)	1.99%
			Harem (https://haremnews.com)	1.01%
			Hengaw (https://hengaw.net)	0.61%
			K24 (www.kurdistan24.net/ckb)	7.54%
			Khaktv (www.khaktv.com)	5.29%
			Millatpress (www.milletpress.com)	5.37%
			Rojnews (https://rojnews.news)	12.97%
			Socialsuli (https://socialsuli.com)	2.01%
			Westga (https://westganews.net)	10.01%
			Wishe (www.wishe.net)	10.01%
			Xelk (https://xelk.org)	39.11%
			Xendan (www.xendan.org)	1.58%

Sport	10000	5	GaliKurdstan (www.gksat.tv)	3.61%
			K24 (www.kurdistan24.net/ckb)	60.31%
			Kurdistantv (https://kurdistantv.net)	24.01%
			Payamtv (www.peyam.net)	2.9%
			Xelk (https://xelk.org)	9.17%

**Table 5:** Number and Percentage of Collected Data according to the channels.

No.	Channels	Total percentage	Categories	Percentage for each class
1	GaliKurdstan (www.gksat.tv)	2.166%	1. Economic	3.61%
			2. Science	3.61%
			3. Sport	3.61%

2	K24 (www.kurdistan24.net/ckb)	29.944%	1. Economic	62.18%
			2. Health	17.91%
			3. Science	1.78%
			4. Social	7.54%
			5. Sport	60.31%

3	Kurdistantv (https://kurdistantv.net)	12.118%	1. Economic	16.85%
			2. Health	12.91%
			3. Science	6.82%
			4. Sport	24.01%

4	Kurdstat (www.kurdsat.tv)	1.138%	1. Economic	3.47%
			2. Health	2.22%

5	Payamtv (www.peyam.net>)	2.390%	1. Economic	2.71%
			2. Health	3.61%
			3. Science	2.73%
			4. Sport	2.9%

6	Rudaw (www.rudaw.net/sorani)	0.026%	1. Economic	0.13%

7	Xendan (www.xendan.org)	2.962%	1. Economic	4.41%
			2. Science	8.82%
			3. Social	1.58%

8	Xelk (https://xelk.org)	12.286%	1. Economic	6.64%
			2. Health	3.51%
			3. Science	3%
			4. Social	39.11%
			5. Sport	9.17%

9	Government (https://gov.krd)	0.308%	1. Health	1.06%
			2. Social	0.48%

10	Jamawar (www.jamawarnews.com)	0.038%	1. Health	0.19%

11	Knnc (www.knnc.net)	0.484%	1. Health	2.42%

12	Kurdiu (www.kurdiu.org/ku)	2.842%	1. Health	14.21%

13	Politic press (https://politicpress.com)	7.484%	1. Health	20.67%
			2. Science	16.75%

14	Rachlaken (www.rachlaken.com)	0.444%	1. Health	2.22%

15	Sharpress (www.sharpress.net)	0.298%	1. Health	1.49%

16	Wishe (www.wishe.net)	4.002%	1. Health	10%
			2. Social	10.01%

17	Xwakurk (www.xwakurk.com)	6.108%	1. Health	7.52%
			2. Science	23.02%

18	Al-ashraq (https://aleshraqtv.iq)	0.624%	1. Science	3.12%

19	Bn24 (https://bn24.org)	0.044%	1. Science	0.22%

20	Chawder (https://chawder.org)	0.182%	1. Science	0.91%

21	Khaktv (www.khaktv.com)	2.164%	1. Science	5.53%
			2. Social	5.29%
22	Kitn (https://kitn.net)	0.686%	1. Science	3.43%

23	Millatpress (www.milletpress.com)	1.552%	1. Science	2.39%
			2. Social	5.37%

24	NRT (www.nrttv.com)	0.938%	1. Science	4.69%

25	Shanpress (www.shanpress.com)	0.198%	1. Science	0.99%

26	Westga (https://westganews.net)	3.336%	1. Science	6.67%
			2. Social	10.01%

27	Zagros (https://zagrosn.com)	1.104%	1. Science	5.52%
28	Awene (https://www.awene.com)	0.362%	1. Social	1.81%

29	Dwaroj (https://dwaroj.net)	0.042%	1. Social	0.21%

30	Gulan (www.gulanmedia.com)	0.398%	1. Social	1.99%

31	Harem (https://haremnews.com)	0.202%	1. Social	1.01%

32	Hengaw (https://hengaw.net)	0.122%	1. Social	0.61%

33	Rojnews (https://rojnews.news)	2.594%	1. Social	12.97%

34	Socialsuli (https://socialsuli.com)	0.402%	1. Social	2.01%

## 4 Experimental Design, Materials and Methods

On the internet, different types of data are available; in this era, the dataset collection is text. For text data gathering, various methods and tools are proposed. The ParsHub tool and the BeautifulSoup library has been used to collect news headlines. The following eight steps should be followed to obtain the data using ParsHub, as shown below:

1. Download: https://www.parsehub.com/quickstart
2. Sign in: using a registered email account
3. New Project: Create a new project for storing the texts
4. Add Link: Click on start project on this URL
5. Select Headlines: Select the headlines on the webpage.
6. Specify PageNnumbers: specify the number of pages you want to collect headlines from them.
7. Get Data: Start collecting the data
8. Export Data: Dataset is exported as a XLSX file format.

**Fig. 1:**
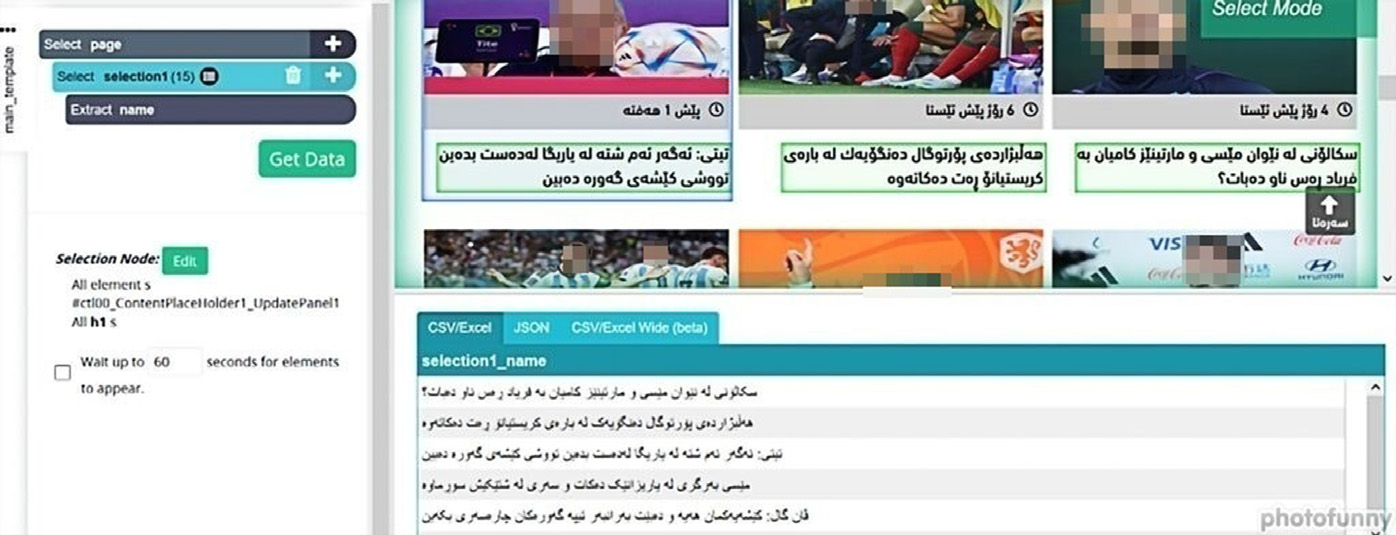
Headline selection.

As shown in the above steps, the first step is installing ParsHbu software for collecting the texts. The second step is signing in with an email account. The third step is creating a new project. The fourth step is adding the URL to the page intended to collect data. The fifth step is selecting some headlines, as shown in Fig. 1. Notably, it is imperative to select three headlines, and the program will automatically select the others within the page based on the researcher's choice.

**Fig. 2:**
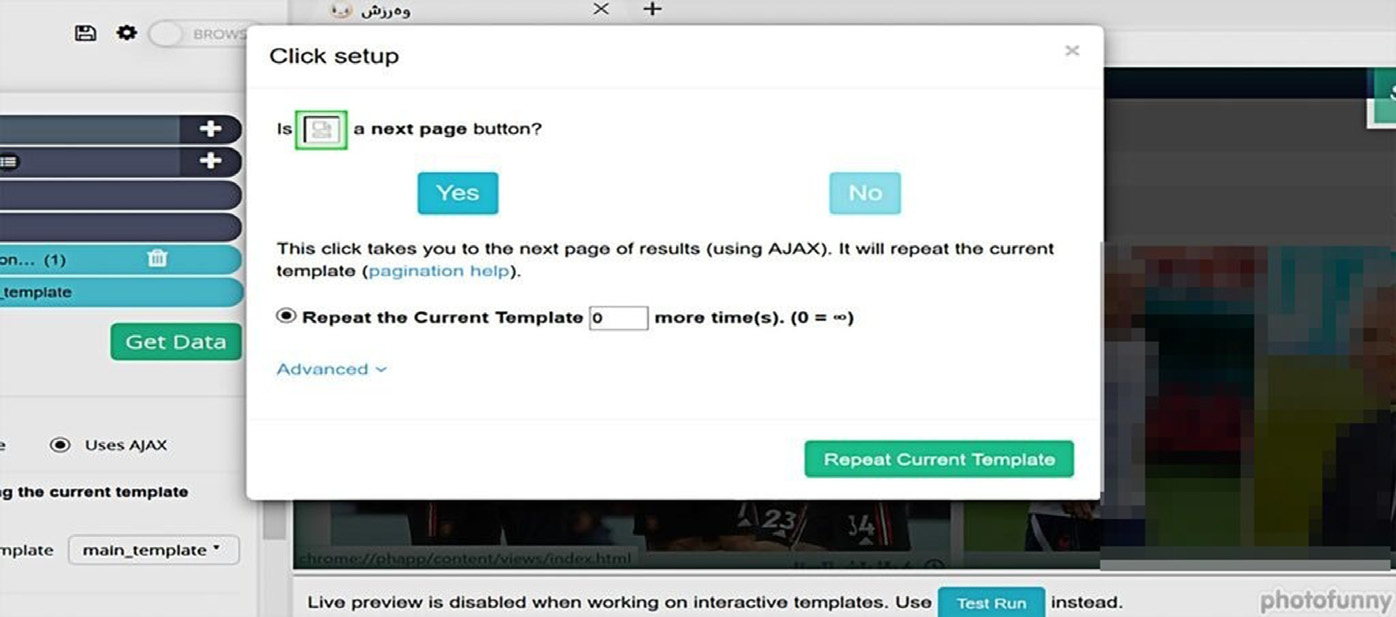
Specifying the number of pages.

In the sixth step, we specify the number of pages from the website we will extract headlines, as shown in Fig. 2. Using this software, users can extract texts from 200 pages on a website.

**Fig. 3:**
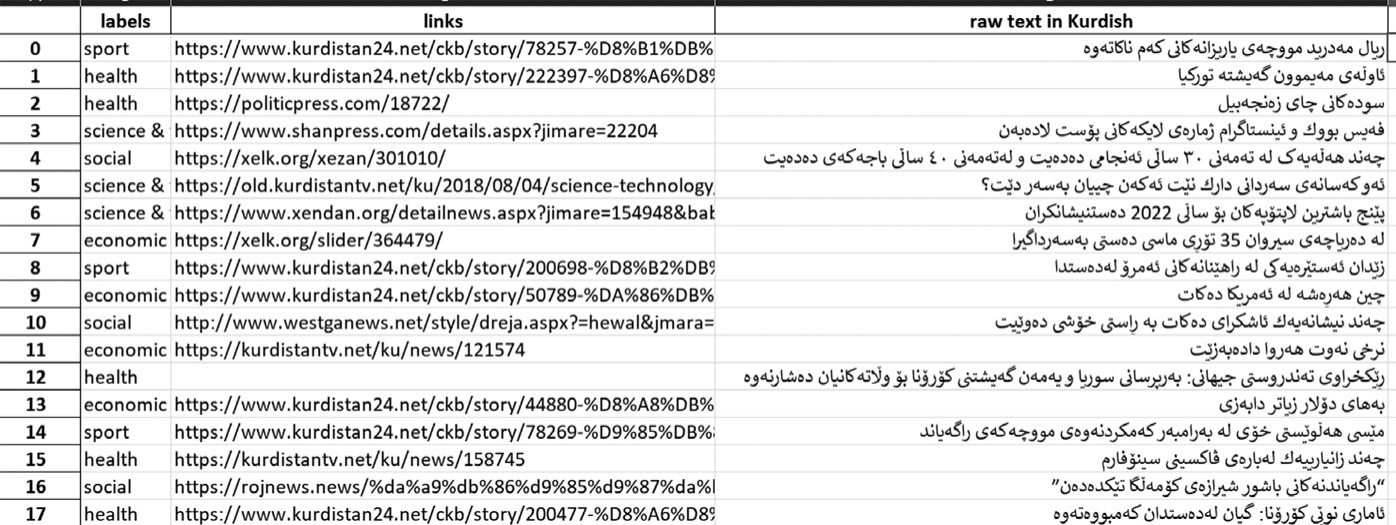
Sample of Corpus in XLSX.

The last step is exporting the headlines as a XLSX file format, as shown in Fig. 3.

Additionally, because ParsHub allows users to extract data several times, we used the BeautifulSoup has been used to crawl the remaining data for the specified dataset. Researchers can use BeautifulSoup's Python library for data collection using the code below. import requests from bs4 import BeautifulSoup link = 'https://www.xendan.org/babetakan.aspx?babet=8&title=ئابوری' r = requests.get(link) soup = BeautifulSoup(r.content, 'html.parser') text = soup.findAll(class_='card-container') texts = [i.texts for i in text] df = pd.DataFrame(np.array(texts), columns=['text']) df.to_csv('data.xlsx)

## 5 Dataset Preprocessing

One of the most important steps after collecting the dataset is preprocessing. In the Kurdish language, the preprocessing steps for Kurdish data were obtained online, includes removing non-Kurdish words, special characters, elongation (letter repetition), symbols, stop words and ineffective numbers. Following that, we tokenized the texts in the dataset. It is crucial pointing out that word tokenization is also challenging due to the nature of the Kurdish language, which is purely morphological. Thus, the language requires its unique tool for performing such tasks. Word tokenization is another process acquired from using KLPT (Kurdish Language Processing Toolkit) [2]. This tool tokenizes Kurdish texts according to the morphological features of the language. The word-tokenization feature helps find the stem of the verbs, as shown in Table 6.

**Table 6:** Sentence-pre-processing.

## 6 Dataset Labeling

**Fig. 4:**
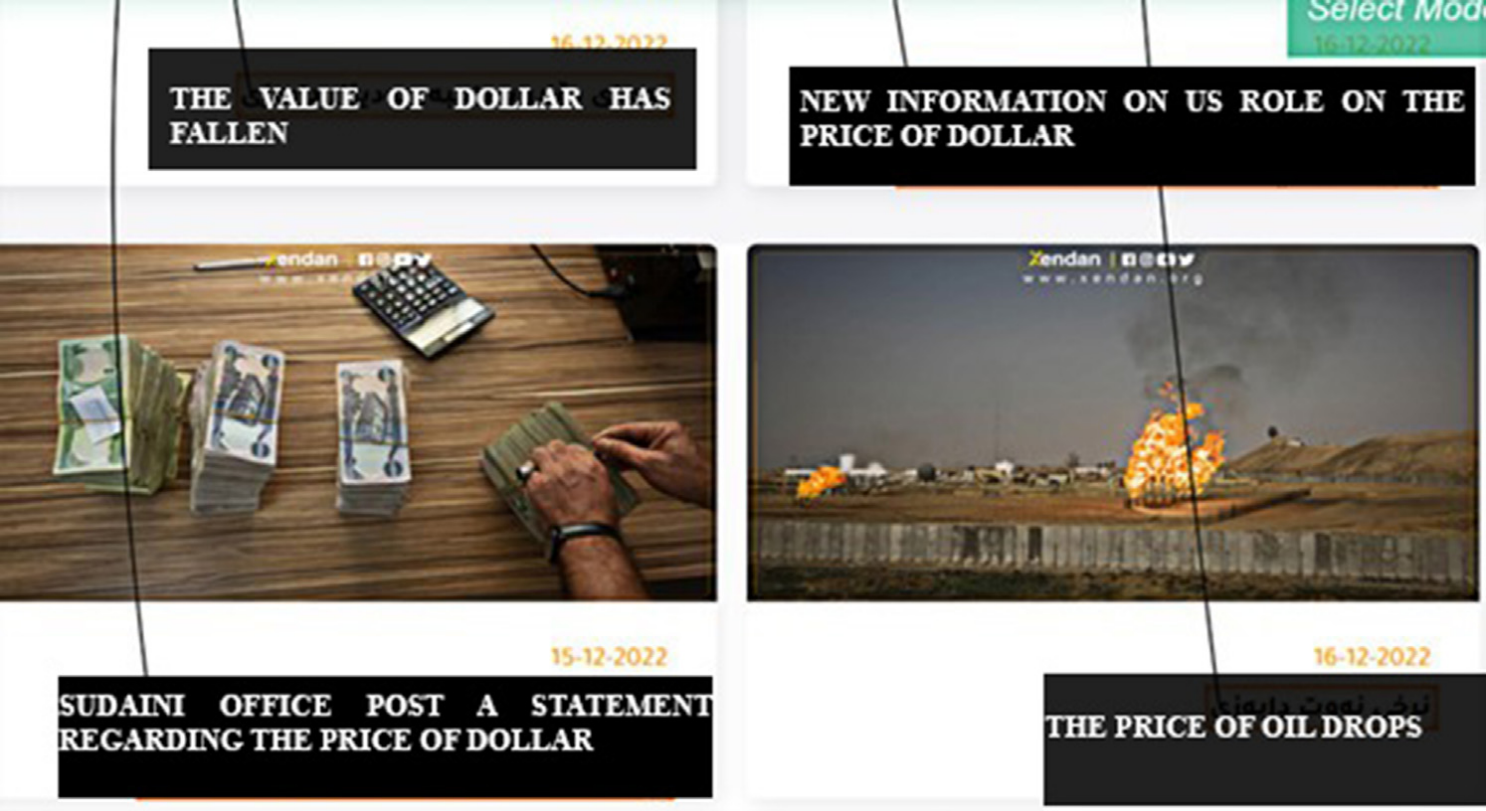
Label selection in the Parshub using node.

Dataset labeling has a significant effect on machine learning and deep learning tools. A dataset can be labeled in three methods. The first method involves reading and understanding texts through human effort. The second method is automatic labeling, which uses pre-trained annotation models to annotate the text. Semi-automatic labeling combines both human and automatic labeling as a third step. In this work, automatic labeling is used for that purpose. Thus, the annotation process is independent of human effort. Due to ParsHub's automatic category extraction, the category in which the news was published can be determined. In other words, it uses the tags written under each news headline, as shown in Fig. 4.

## Ethics Statement

This manuscript contains data acquired by using two web scraping tools. Regarding, the Terms of service (ToS), all web resources listed in Table 4 and used in the dataset allow for scraping and distributing data. Due to the fact that Kurdish news websites are free and open to everyone, thus, allowing articles to be scrapped. We confirm that data are not used for any fraudulent purposes, such as making profits (e.g., business), DDoS, data theft, or any other bad intentions. Regarding copyright, news reports are published on public news websites that can be accessed easily by anyone who has access to the Internet, and It is similar to search engines which use bots to index Web pages. It is important to note that the data in this article belong to the news websites. In this dataset, the privacy rights of individuals are protected. Even though the data is free and available to everyone, we have removed each website's identity (Uniform Resource Locator URL). The data collection process did not involve the collection of personal information. We removed identities from the dataset if they appeared in it. The dataset was neutralized according to legal and ethical guidelines and policies. The purpose of this task is to build a dataset that can classify news texts into multiple classes, not to target users or channels or any political parties. The data in the dataset were obtained from publicly available news channels. There is no scraping of data directly from social media platforms (such as Twitter and Facebook). Thus, it does not violate their scrapping policies.

## CRediT authorship contribution statement

**Soran Badawi:** Supervision, Data curation, Conceptualization, Methodology, Visualization, Project administration, Funding acquisition, Writing – original draft, Writing – review & editing. **Ari M. Saeed:** Software, Formal analysis, Investigation, Resources, Supervision, Validation, Writing – review & editing. **Sara A. Ahmed:** Software, Writing – review & editing. **Peshraw Ahmed Abdalla:** Software, Formal analysis, Investigation, Resources. **Diyari A. Hassan:** Validation, Writing – review & editing.

## Declaration of Competing Interest

The authors declare that the work described in this article has not been influenced by competing financial interests or personal relationships.

## Data Availability

Kurdish News Dataset Headlines (KNDH) through Multiclass Classificatio (Original data) (Mendeley Data).
